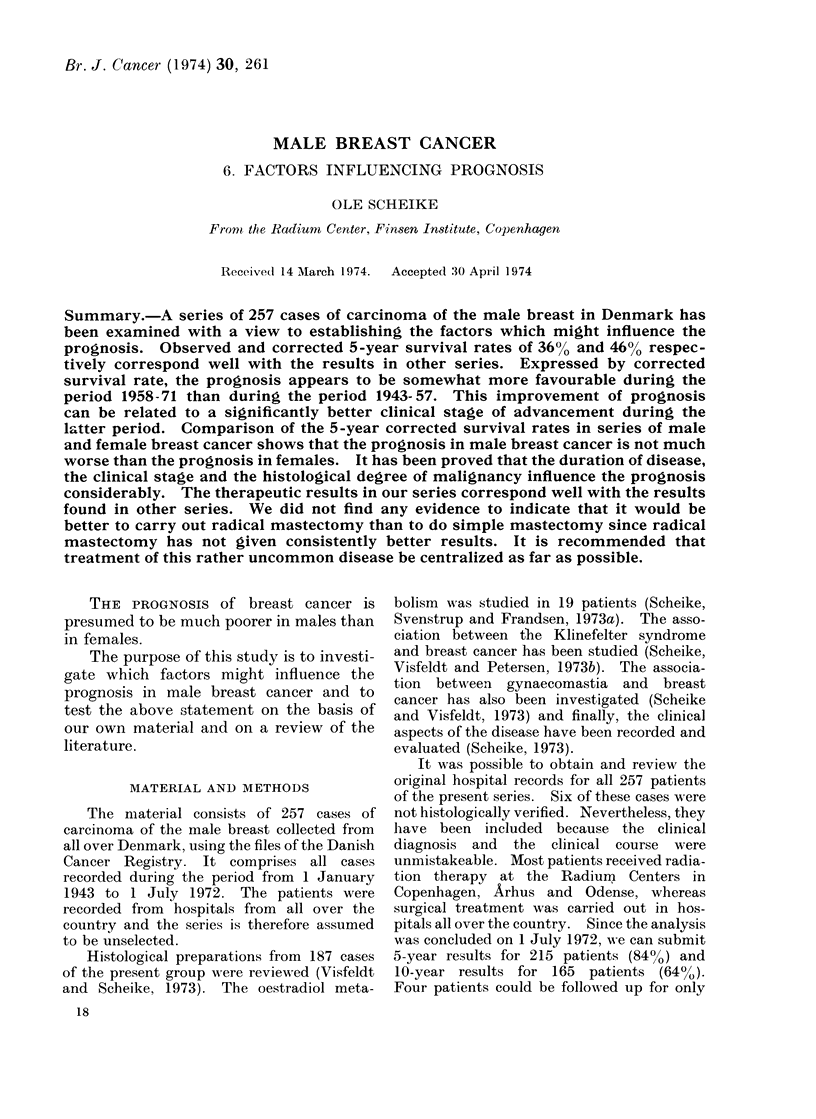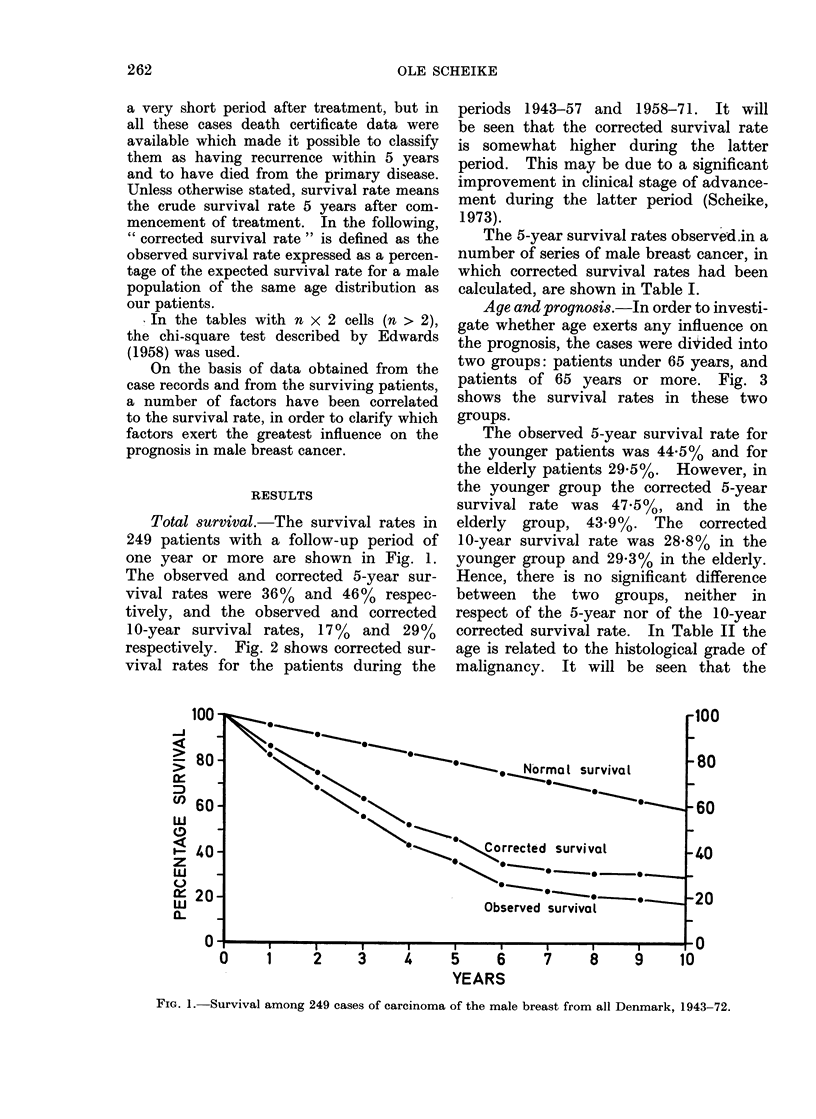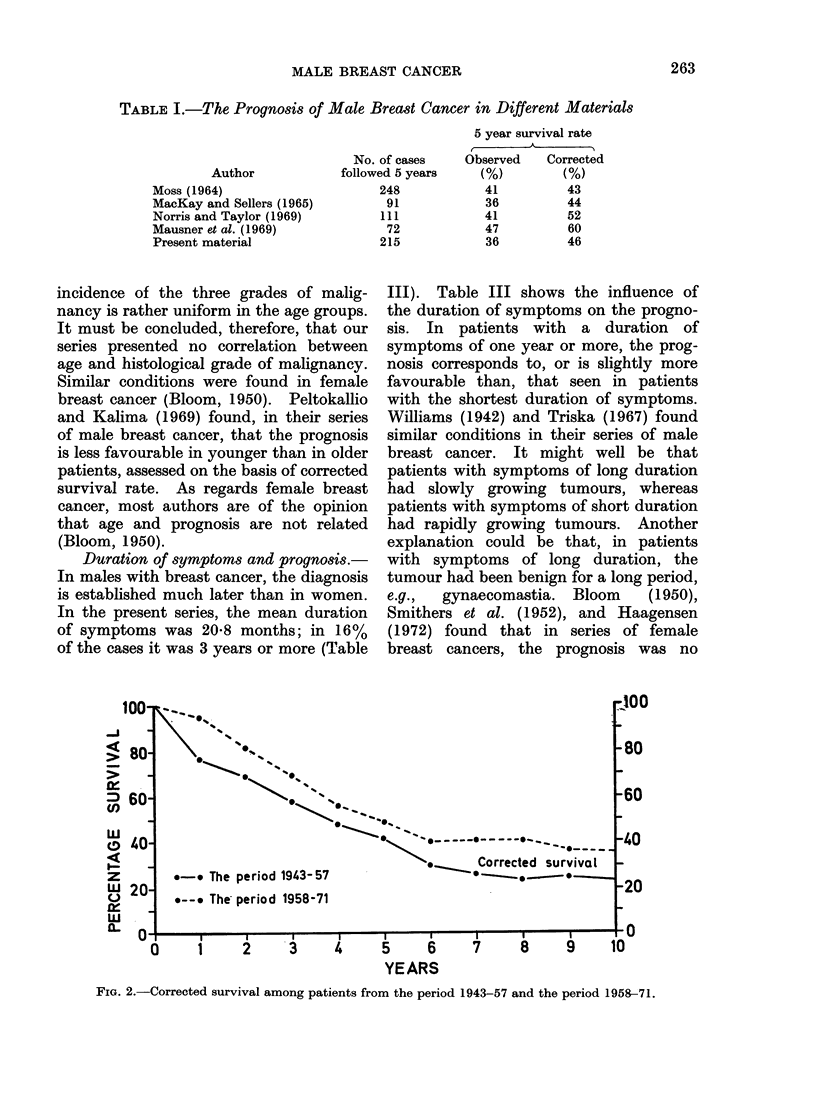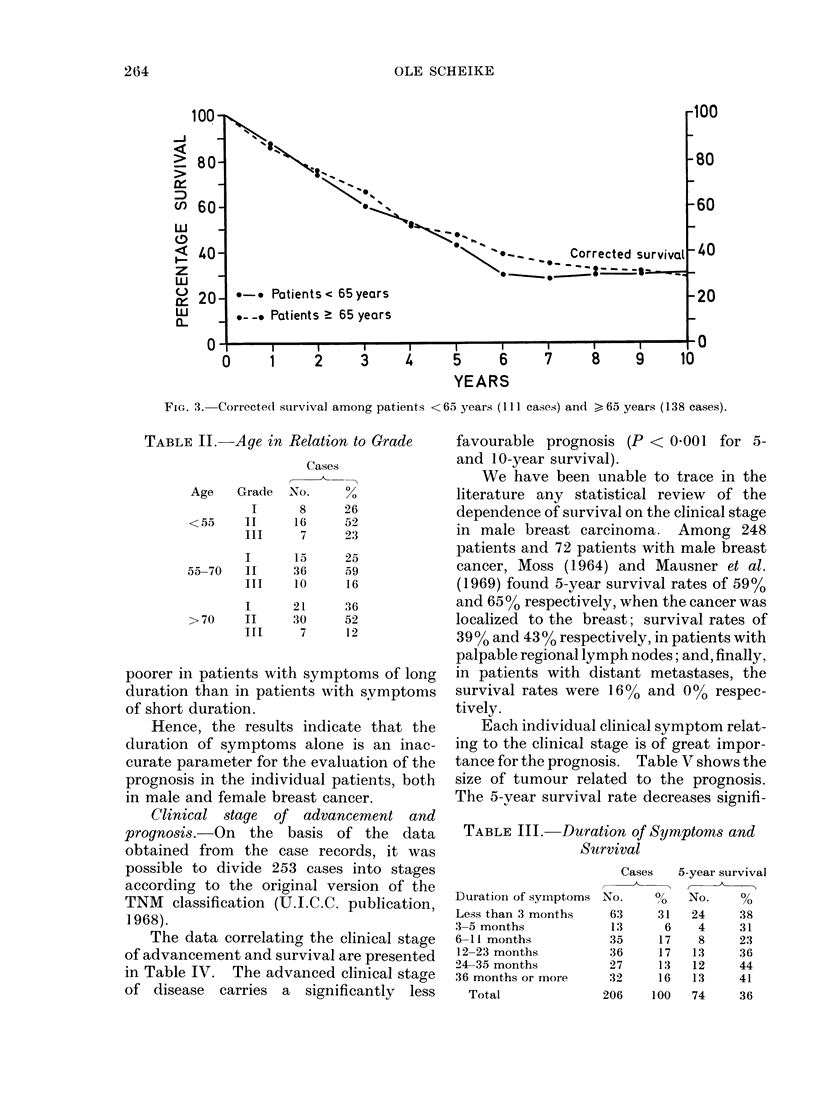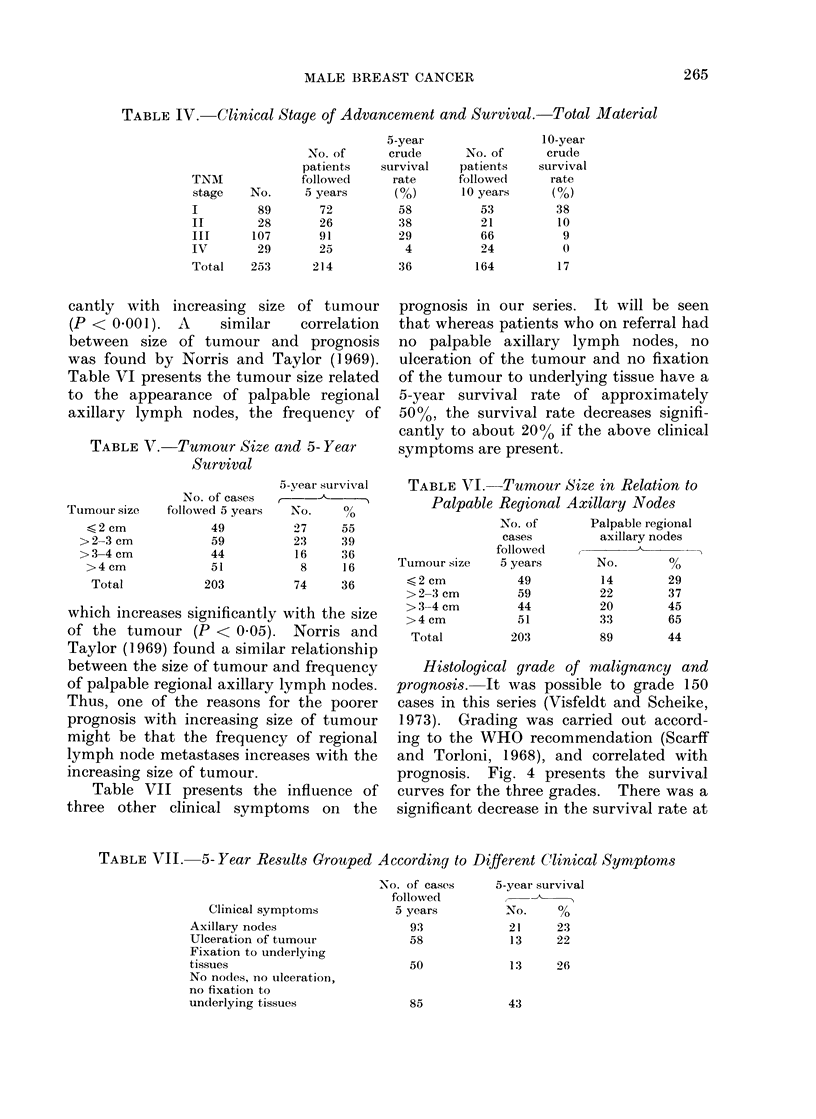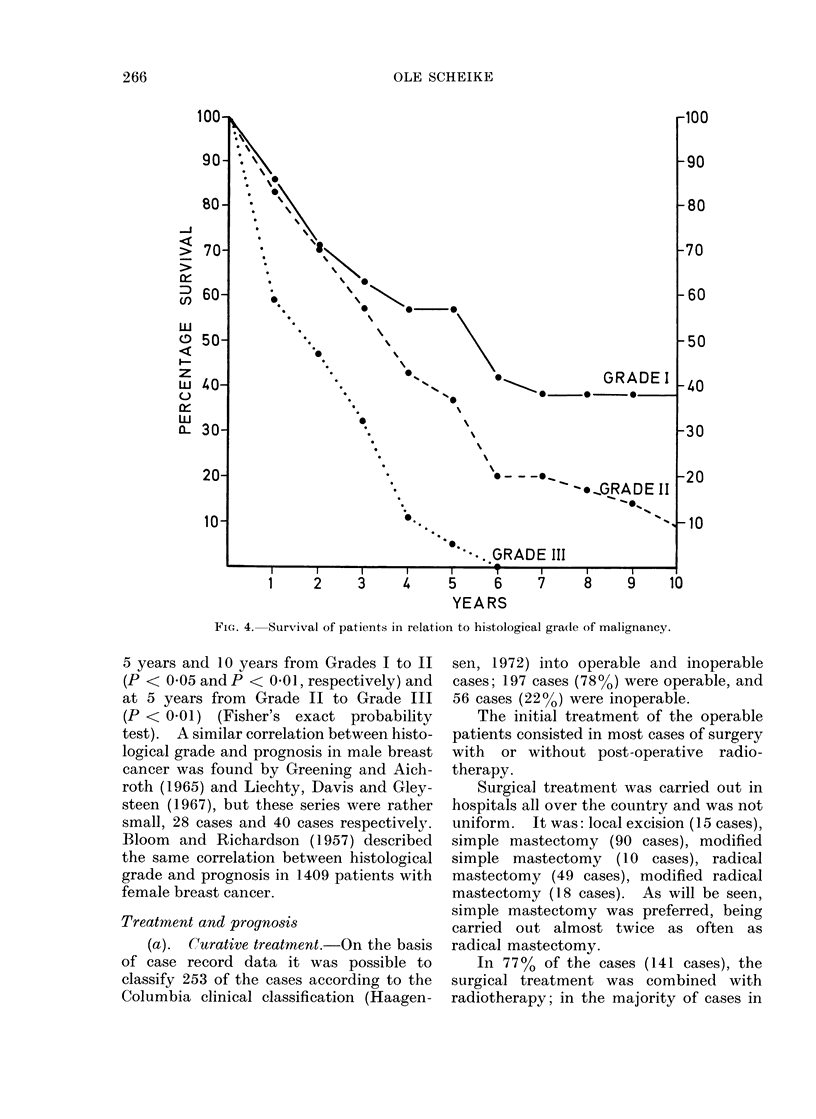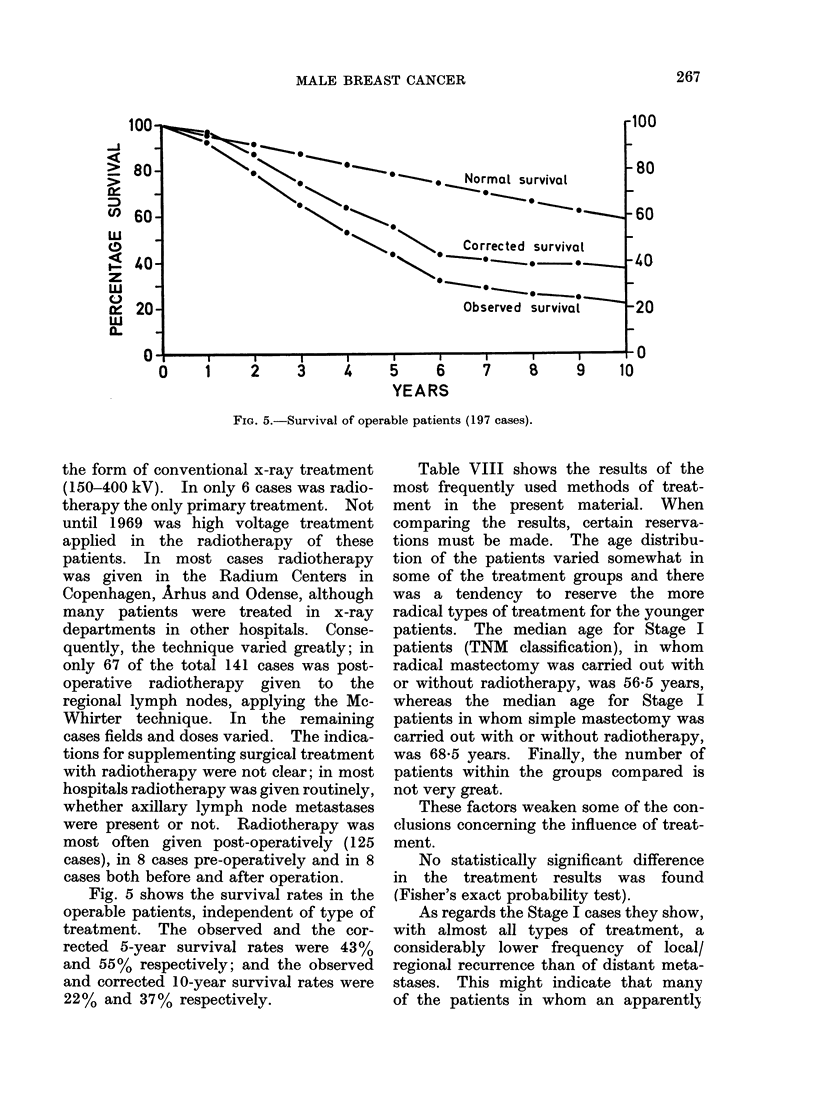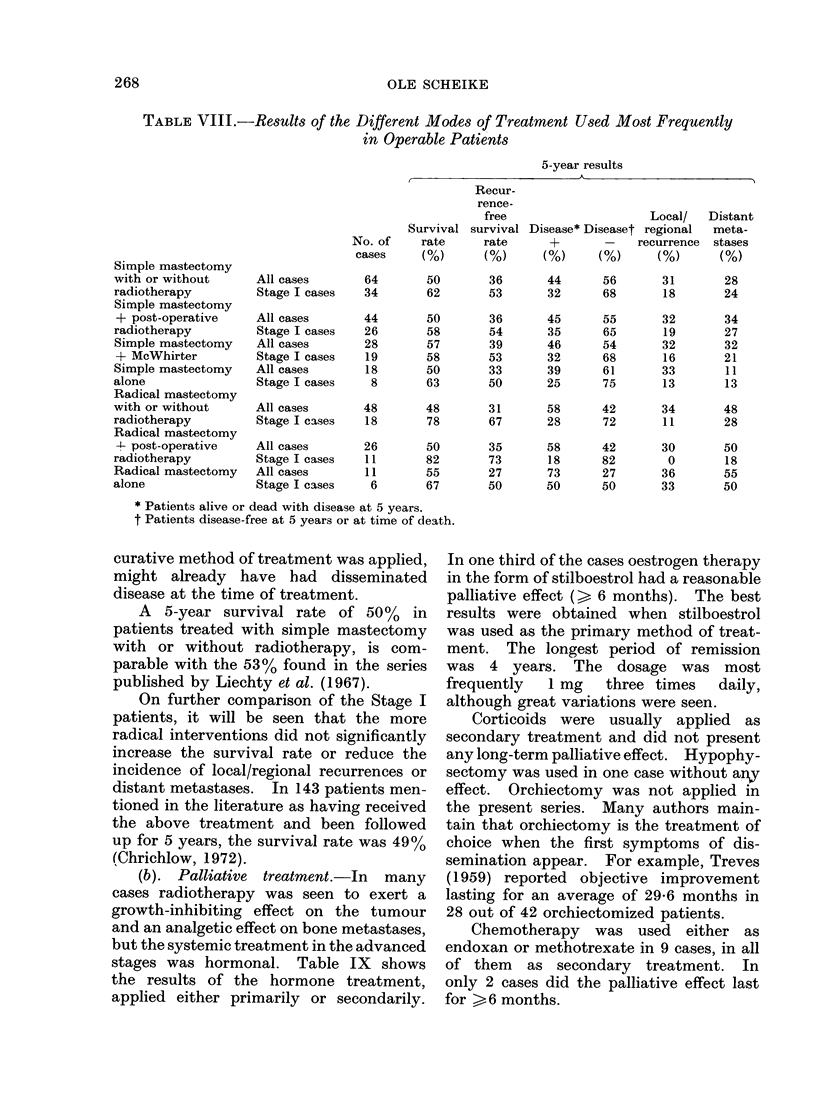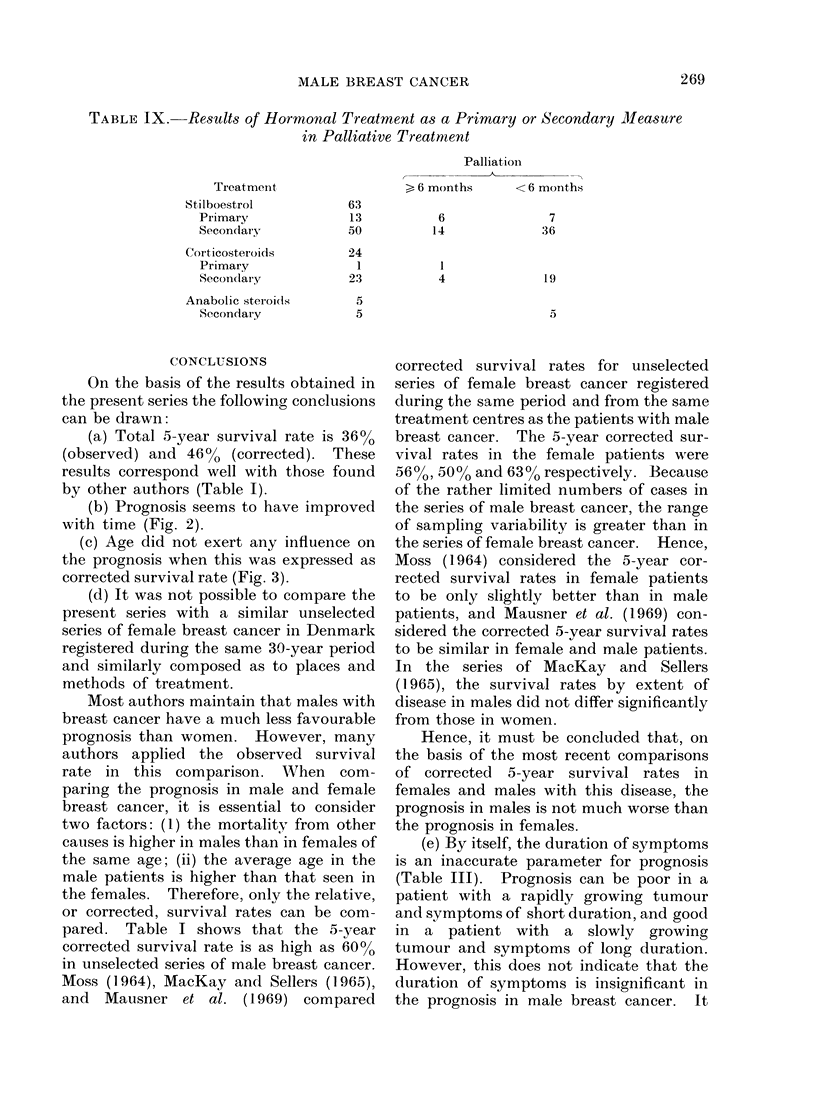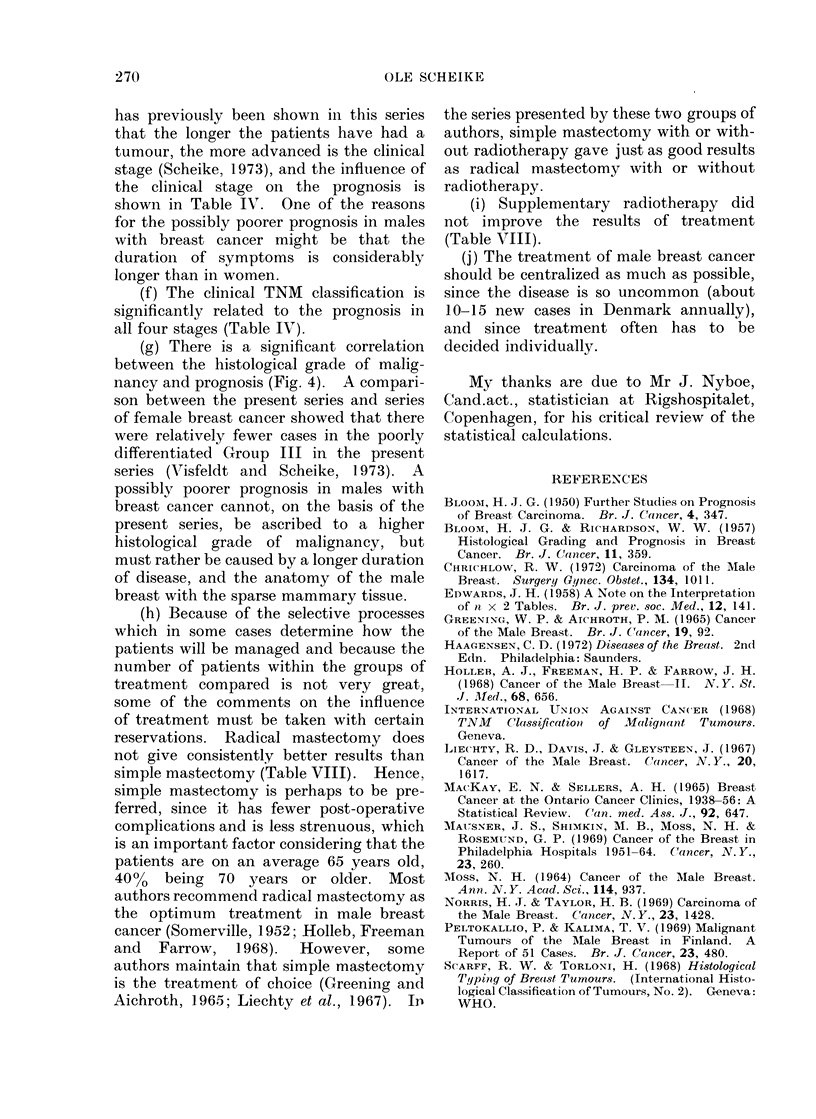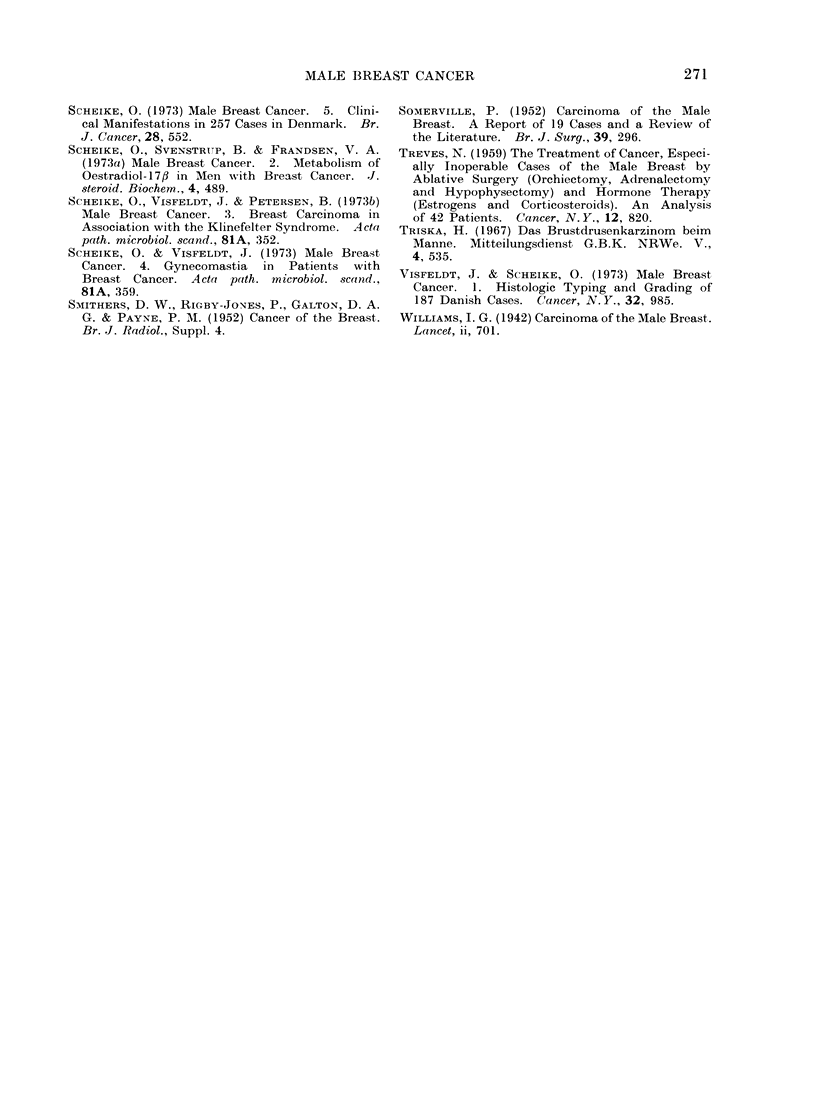# Male Breast Cancer

**DOI:** 10.1038/bjc.1974.190

**Published:** 1974-09

**Authors:** Ole Scheike

## Abstract

A series of 257 cases of carcinoma of the male breast in Denmark has been examined with a view to establishing the factors which might influence the prognosis. Observed and corrected 5-year survival rates of 36% and 46% respectively correspond well with the results in other series. Expressed by corrected survival rate, the prognosis appears to be somewhat more favourable during the period 1958-71 than during the period 1943-57. This improvement of prognosis can be related to a significantly better clinical stage of advancement during the latter period. Comparison of the 5-year corrected survival rates in series of male and female breast cancer shows that the prognosis in male breast cancer is not much worse than the prognosis in females. It has been proved that the duration of disease, the clinical stage and the histological degree of malignancy influence the prognosis considerably. The therapeutic results in our series correspond well with the results found in other series. We did not find any evidence to indicate that it would be better to carry out radical mastectomy than to do simple mastectomy since radical mastectomy has not given consistently better results. It is recommended that treatment of this rather uncommon disease be centralized as far as possible.


*Br. J. Cancer* (1974) **30**, 261

### MALE BREAST CANCER

### 6. FACTORS INFLUENCING PROGNOSIS

OLE SCHEIKE

*From the Radium Center, Finsen Institute, Copenhagen*

Received 14 March 1974. Accepted 30 April 1974

**Summary.—A series of 257 cases of carcinoma of the male breast in Denmark has been examined with a view to establishing the factors which might influence the prognosis. Observed and corrected 5-year survival rates of 36% and 46% respectively correspond well with the results in other series. Expressed by corrected survival rate, the prognosis appears to be somewhat more favourable during the period 1958-71 than during the period 1943-57. This improvement of prognosis can be related to a significantly better clinical stage of advancement during the latter period. Comparison of the 5-year corrected survival rates in series of male and female breast cancer shows that the prognosis in male breast cancer is not much worse than the prognosis in females. It has been proved that the duration of disease, the clinical stage and the histological degree of malignancy influence the prognosis considerably. The therapeutic results in our series correspond well with the results found in other series. We did not find any evidence to indicate that it would be better to carry out radical mastectomy than to do simple mastectomy since radical mastectomy has not given consistently better results. It is recommended that treatment of this rather uncommon disease be centralized as far as possible.**

THE PROGNOSIS of breast cancer is presumed to be much poorer in males than in females.

The purpose of this study is to investigate which factors might influence the prognosis in male breast cancer and to test the above statement on the basis of our own material and on a review of the literature.

### MATERIAL AND METHODS

The material consists of 257 cases of carcinoma of the male breast collected from all over Denmark, using the files of the Danish Cancer Registry. It comprises all cases recorded during the period from 1 January 1943 to 1 July 1972. The patients were recorded from hospitals from all over the country and the series is therefore assumed to be unselected.

Histological preparations from 187 cases of the present group were reviewed (Visfeldt and Scheike, 1973). The oestradiol metabolism was studied in 19 patients (Scheike, Svenstrup and Frandsen, 1973*a*). The association between the Klinefelter syndrome and breast cancer has been studied (Scheike, Visfeldt and Petersen, 1973*b*). The association between gynaecomastia and breast cancer has also been investigated (Scheike and Visfeldt, 1973) and finally, the clinical aspects of the disease have been recorded and evaluated (Scheike, 1973).

It was possible to obtain and review the original hospital records for all 257 patients of the present series. Six of these cases were not histologically verified. Nevertheless, they have been included because the clinical diagnosis and the clinical course were unmistakeable. Most patients received radiation therapy at the Radium Centers in Copenhagen, Århus and Odense, whereas surgical treatment was carried out in hospitals all over the country. Since the analysis was concluded on 1 July 1972, we can submit 5-year results for 215 patients (84%) and 10-year results for 165 patients (64%). Four patients could be followed up for only a very short period after treatment, but in all these cases death certificate data were available which made it possible to classify them as having recurrence within 5 years and to have died from the primary disease. Unless otherwise stated, survival rate means the crude survival rate 5 years after commencement of treatment. In the following, " corrected survival rate " is defined as the observed survival rate expressed as a percentage of the expected survival rate for a male population of the same age distribution as our patients.

In the tables with $n \times 2$ cells ($n > 2$), the chi-square test described by Edwards (1958) was used.

On the basis of data obtained from the case records and from the surviving patients, a number of factors have been correlated to the survival rate, in order to clarify which factors exert the greatest influence on the prognosis in male breast cancer.

### RESULTS

*Total survival.*—The survival rates in 249 patients with a follow-up period of one year or more are shown in Fig. 1. The observed and corrected 5-year survival rates were 36% and 46% respectively, and the observed and corrected 10-year survival rates, 17% and 29% respectively. Fig. 2 shows corrected survival rates for the patients during the periods 1943–57 and 1958–71. It will be seen that the corrected survival rate is somewhat higher during the latter period. This may be due to a significant improvement in clinical stage of advancement during the latter period (Scheike, 1973).

The 5-year survival rates observed in a number of series of male breast cancer, in which corrected survival rates had been calculated, are shown in Table I.

*Age and prognosis.*—In order to investigate whether age exerts any influence on the prognosis, the cases were divided into two groups: patients under 65 years, and patients of 65 years or more. Fig. 3 shows the survival rates in these two groups.

The observed 5-year survival rate for the younger patients was 44·5% and for the elderly patients 29·5%. However, in the younger group the corrected 5-year survival rate was 47·5%, and in the elderly group, 43·9%. The corrected 10-year survival rate was 28·8% in the younger group and 29·3% in the elderly. Hence, there is no significant difference between the two groups, neither in respect of the 5-year nor of the 10-year corrected survival rate. In Table II the age is related to the histological grade of malignancy. It will be seen that the

FIG. 1.—Survival among 249 cases of carcinoma of the male breast from all Denmark, 1943–72.

TABLE I.—*The Prognosis of Male Breast Cancer in Different Materials*

| Author | No. of cases followed 5 years | 5 year survival rate Observed (%) | Corrected (%) |
|---|---|---|---|
| Moss (1964) | 248 | 41 | 43 |
| MacKay and Sellers (1965) | 91 | 36 | 44 |
| Norris and Taylor (1969) | 111 | 41 | 52 |
| Mausner *et al.* (1969) | 72 | 47 | 60 |
| Present material | 215 | 36 | 46 |

incidence of the three grades of malignancy is rather uniform in the age groups. It must be concluded, therefore, that our series presented no correlation between age and histological grade of malignancy. Similar conditions were found in female breast cancer (Bloom, 1950). Peltokallio and Kalima (1969) found, in their series of male breast cancer, that the prognosis is less favourable in younger than in older patients, assessed on the basis of corrected survival rate. As regards female breast cancer, most authors are of the opinion that age and prognosis are not related (Bloom, 1950).

*Duration of symptoms and prognosis.*—In males with breast cancer, the diagnosis is established much later than in women. In the present series, the mean duration of symptoms was 20·8 months; in 16% of the cases it was 3 years or more (Table III). Table III shows the influence of the duration of symptoms on the prognosis. In patients with a duration of symptoms of one year or more, the prognosis corresponds to, or is slightly more favourable than, that seen in patients with the shortest duration of symptoms. Williams (1942) and Triska (1967) found similar conditions in their series of male breast cancer. It might well be that patients with symptoms of long duration had slowly growing tumours, whereas patients with symptoms of short duration had rapidly growing tumours. Another explanation could be that, in patients with symptoms of long duration, the tumour had been benign for a long period, *e.g.*, gynaecomastia. Bloom (1950), Smithers *et al.* (1952), and Haagensen (1972) found that in series of female breast cancers, the prognosis was no

FIG. 2.—Corrected survival among patients from the period 1943–57 and the period 1958–71.

FIG. 3.—Corrected survival among patients <65 years (111 cases) and ≥65 years (138 cases).

TABLE II.—*Age in Relation to Grade*

| Age | Grade | Cases No. | Cases % |
|---|---|---|---|
| | I | 8 | 26 |
| <55 | II | 16 | 52 |
| | III | 7 | 23 |
| | I | 15 | 25 |
| 55–70 | II | 36 | 59 |
| | III | 10 | 16 |
| | I | 21 | 36 |
| >70 | II | 30 | 52 |
| | III | 7 | 12 |

poorer in patients with symptoms of long duration than in patients with symptoms of short duration.

Hence, the results indicate that the duration of symptoms alone is an inaccurate parameter for the evaluation of the prognosis in the individual patients, both in male and female breast cancer.

*Clinical stage of advancement and prognosis.*—On the basis of the data obtained from the case records, it was possible to divide 253 cases into stages according to the original version of the TNM classification (U.I.C.C. publication, 1968).

The data correlating the clinical stage of advancement and survival are presented in Table IV. The advanced clinical stage of disease carries a significantly less favourable prognosis ($P < 0{\cdot}001$ for 5- and 10-year survival).

We have been unable to trace in the literature any statistical review of the dependence of survival on the clinical stage in male breast carcinoma. Among 248 patients and 72 patients with male breast cancer, Moss (1964) and Mausner *et al.* (1969) found 5-year survival rates of 59% and 65% respectively, when the cancer was localized to the breast; survival rates of 39% and 43% respectively, in patients with palpable regional lymph nodes; and, finally, in patients with distant metastases, the survival rates were 16% and 0% respectively.

Each individual clinical symptom relating to the clinical stage is of great importance for the prognosis. Table V shows the size of tumour related to the prognosis. The 5-year survival rate decreases signifi-

TABLE III.—*Duration of Symptoms and Survival*

| Duration of symptoms | Cases No. | Cases % | 5-year survival No. | 5-year survival % |
|---|---|---|---|---|
| Less than 3 months | 63 | 31 | 24 | 38 |
| 3–5 months | 13 | 6 | 4 | 31 |
| 6–11 months | 35 | 17 | 8 | 23 |
| 12–23 months | 36 | 17 | 13 | 36 |
| 24–35 months | 27 | 13 | 12 | 44 |
| 36 months or more | 32 | 16 | 13 | 41 |
| Total | 206 | 100 | 74 | 36 |

TABLE IV.—*Clinical Stage of Advancement and Survival.—Total Material*

| TNM stage | No. | No. of patients followed 5 years | 5-year crude survival rate (%) | No. of patients followed 10 years | 10-year crude survival rate (%) |
|---|---|---|---|---|---|
| I | 89 | 72 | 58 | 53 | 38 |
| II | 28 | 26 | 38 | 21 | 10 |
| III | 107 | 91 | 29 | 66 | 9 |
| IV | 29 | 25 | 4 | 24 | 0 |
| Total | 253 | 214 | 36 | 164 | 17 |

cantly with increasing size of tumour ($P < 0{\cdot}001$). A similar correlation between size of tumour and prognosis was found by Norris and Taylor (1969). Table VI presents the tumour size related to the appearance of palpable regional axillary lymph nodes, the frequency of which increases significantly with the size of the tumour ($P < 0{\cdot}05$). Norris and Taylor (1969) found a similar relationship between the size of tumour and frequency of palpable regional axillary lymph nodes. Thus, one of the reasons for the poorer prognosis with increasing size of tumour might be that the frequency of regional lymph node metastases increases with the increasing size of tumour.

TABLE V.—*Tumour Size and 5-Year Survival*

| Tumour size | No. of cases followed 5 years | 5-year survival No. | % |
|---|---|---|---|
| ≤2 cm | 49 | 27 | 55 |
| >2–3 cm | 59 | 23 | 39 |
| >3–4 cm | 44 | 16 | 36 |
| >4 cm | 51 | 8 | 16 |
| Total | 203 | 74 | 36 |

Table VII presents the influence of three other clinical symptoms on the prognosis in our series. It will be seen that whereas patients who on referral had no palpable axillary lymph nodes, no ulceration of the tumour and no fixation of the tumour to underlying tissue have a 5-year survival rate of approximately 50%, the survival rate decreases significantly to about 20% if the above clinical symptoms are present.

TABLE VI.—*Tumour Size in Relation to Palpable Regional Axillary Nodes*

| Tumour size | No. of cases followed 5 years | Palpable regional axillary nodes No. | % |
|---|---|---|---|
| ≤2 cm | 49 | 14 | 29 |
| >2–3 cm | 59 | 22 | 37 |
| >3–4 cm | 44 | 20 | 45 |
| >4 cm | 51 | 33 | 65 |
| Total | 203 | 89 | 44 |

*Histological grade of malignancy and prognosis.*—It was possible to grade 150 cases in this series (Visfeldt and Scheike, 1973). Grading was carried out according to the WHO recommendation (Scarff and Torloni, 1968), and correlated with prognosis. Fig. 4 presents the survival curves for the three grades. There was a significant decrease in the survival rate at

TABLE VII.—*5-Year Results Grouped According to Different Clinical Symptoms*

| Clinical symptoms | No. of cases followed 5 years | 5-year survival No. | % |
|---|---|---|---|
| Axillary nodes | 93 | 21 | 23 |
| Ulceration of tumour | 58 | 13 | 22 |
| Fixation to underlying tissues | 50 | 13 | 26 |
| No nodes, no ulceration, no fixation to underlying tissues | 85 | 43 | |

FIG. 4.—Survival of patients in relation to histological grade of malignancy.

5 years and 10 years from Grades I to II ($P < 0{\cdot}05$ and $P < 0{\cdot}01$, respectively) and at 5 years from Grade II to Grade III ($P < 0{\cdot}01$) (Fisher's exact probability test). A similar correlation between histological grade and prognosis in male breast cancer was found by Greening and Aichroth (1965) and Liechty, Davis and Gleysteen (1967), but these series were rather small, 28 cases and 40 cases respectively. Bloom and Richardson (1957) described the same correlation between histological grade and prognosis in 1409 patients with female breast cancer.

*Treatment and prognosis*

(*a*). *Curative treatment.*—On the basis of case record data it was possible to classify 253 of the cases according to the Columbia clinical classification (Haagensen, 1972) into operable and inoperable cases; 197 cases (78%) were operable, and 56 cases (22%) were inoperable.

The initial treatment of the operable patients consisted in most cases of surgery with or without post-operative radiotherapy.

Surgical treatment was carried out in hospitals all over the country and was not uniform. It was: local excision (15 cases), simple mastectomy (90 cases), modified simple mastectomy (10 cases), radical mastectomy (49 cases), modified radical mastectomy (18 cases). As will be seen, simple mastectomy was preferred, being carried out almost twice as often as radical mastectomy.

In 77% of the cases (141 cases), the surgical treatment was combined with radiotherapy; in the majority of cases in

FIG. 5.—Survival of operable patients (197 cases).

the form of conventional x-ray treatment (150–400 kV). In only 6 cases was radiotherapy the only primary treatment. Not until 1969 was high voltage treatment applied in the radiotherapy of these patients. In most cases radiotherapy was given in the Radium Centers in Copenhagen, Århus and Odense, although many patients were treated in x-ray departments in other hospitals. Consequently, the technique varied greatly; in only 67 of the total 141 cases was post-operative radiotherapy given to the regional lymph nodes, applying the McWhirter technique. In the remaining cases fields and doses varied. The indications for supplementing surgical treatment with radiotherapy were not clear; in most hospitals radiotherapy was given routinely, whether axillary lymph node metastases were present or not. Radiotherapy was most often given post-operatively (125 cases), in 8 cases pre-operatively and in 8 cases both before and after operation.

Fig. 5 shows the survival rates in the operable patients, independent of type of treatment. The observed and the corrected 5-year survival rates were 43% and 55% respectively; and the observed and corrected 10-year survival rates were 22% and 37% respectively.

Table VIII shows the results of the most frequently used methods of treatment in the present material. When comparing the results, certain reservations must be made. The age distribution of the patients varied somewhat in some of the treatment groups and there was a tendency to reserve the more radical types of treatment for the younger patients. The median age for Stage I patients (TNM classification), in whom radical mastectomy was carried out with or without radiotherapy, was 56·5 years, whereas the median age for Stage I patients in whom simple mastectomy was carried out with or without radiotherapy, was 68·5 years. Finally, the number of patients within the groups compared is not very great.

These factors weaken some of the conclusions concerning the influence of treatment.

No statistically significant difference in the treatment results was found (Fisher's exact probability test).

As regards the Stage I cases they show, with almost all types of treatment, a considerably lower frequency of local/regional recurrence than of distant metastases. This might indicate that many of the patients in whom an apparently

TABLE VIII.—*Results of the Different Modes of Treatment Used Most Frequently in Operable Patients*

| | | | 5-year results | | | | | |
|---|---|---|---|---|---|---|---|---|
| | | No. of cases | Survival rate (%) | Recurrence-free survival rate (%) | Disease* + (%) | Disease† − (%) | Local/ regional recurrence (%) | Distant metastases (%) |
| Simple mastectomy with or without radiotherapy | All cases | 64 | 50 | 36 | 44 | 56 | 31 | 28 |
| | Stage I cases | 34 | 62 | 53 | 32 | 68 | 18 | 24 |
| Simple mastectomy + post-operative radiotherapy | All cases | 44 | 50 | 36 | 45 | 55 | 32 | 34 |
| | Stage I cases | 26 | 58 | 54 | 35 | 65 | 19 | 27 |
| Simple mastectomy + McWhirter | All cases | 28 | 57 | 39 | 46 | 54 | 32 | 32 |
| | Stage I cases | 19 | 58 | 53 | 32 | 68 | 16 | 21 |
| Simple mastectomy alone | All cases | 18 | 50 | 33 | 39 | 61 | 33 | 11 |
| | Stage I cases | 8 | 63 | 50 | 25 | 75 | 13 | 13 |
| Radical mastectomy with or without radiotherapy | All cases | 48 | 48 | 31 | 58 | 42 | 34 | 48 |
| | Stage I cases | 18 | 78 | 67 | 28 | 72 | 11 | 28 |
| Radical mastectomy + post-operative radiotherapy | All cases | 26 | 50 | 35 | 58 | 42 | 30 | 50 |
| | Stage I cases | 11 | 82 | 73 | 18 | 82 | 0 | 18 |
| Radical mastectomy alone | All cases | 11 | 55 | 27 | 73 | 27 | 36 | 55 |
| | Stage I cases | 6 | 67 | 50 | 50 | 50 | 33 | 50 |

* Patients alive or dead with disease at 5 years.

† Patients disease-free at 5 years or at time of death.

curative method of treatment was applied, might already have had disseminated disease at the time of treatment.

A 5-year survival rate of 50% in patients treated with simple mastectomy with or without radiotherapy, is comparable with the 53% found in the series published by Liechty *et al.* (1967).

On further comparison of the Stage I patients, it will be seen that the more radical interventions did not significantly increase the survival rate or reduce the incidence of local/regional recurrences or distant metastases. In 143 patients mentioned in the literature as having received the above treatment and been followed up for 5 years, the survival rate was 49% (Chrichlow, 1972).

(*b*). *Palliative treatment*.—In many cases radiotherapy was seen to exert a growth-inhibiting effect on the tumour and an analgetic effect on bone metastases, but the systemic treatment in the advanced stages was hormonal. Table IX shows the results of the hormone treatment, applied either primarily or secondarily. In one third of the cases oestrogen therapy in the form of stilboestrol had a reasonable palliative effect ($\geqslant$ 6 months). The best results were obtained when stilboestrol was used as the primary method of treatment. The longest period of remission was 4 years. The dosage was most frequently 1 mg three times daily, although great variations were seen.

Corticoids were usually applied as secondary treatment and did not present any long-term palliative effect. Hypophysectomy was used in one case without any effect. Orchiectomy was not applied in the present series. Many authors maintain that orchiectomy is the treatment of choice when the first symptoms of dissemination appear. For example, Treves (1959) reported objective improvement lasting for an average of 29·6 months in 28 out of 42 orchiectomized patients.

Chemotherapy was used either as endoxan or methotrexate in 9 cases, in all of them as secondary treatment. In only 2 cases did the palliative effect last for $\geqslant$6 months.

TABLE IX.—*Results of Hormonal Treatment as a Primary or Secondary Measure in Palliative Treatment*

| Treatment | | Palliation ≥6 months | Palliation <6 months |
|---|---|---|---|
| Stilboestrol | 63 | | |
| Primary | 13 | 6 | 7 |
| Secondary | 50 | 14 | 36 |
| Corticosteroids | 24 | | |
| Primary | 1 | 1 | |
| Secondary | 23 | 4 | 19 |
| Anabolic steroids | 5 | | |
| Secondary | 5 | | 5 |

### CONCLUSIONS

On the basis of the results obtained in the present series the following conclusions can be drawn:

(a) Total 5-year survival rate is 36% (observed) and 46% (corrected). These results correspond well with those found by other authors (Table I).

(b) Prognosis seems to have improved with time (Fig. 2).

(c) Age did not exert any influence on the prognosis when this was expressed as corrected survival rate (Fig. 3).

(d) It was not possible to compare the present series with a similar unselected series of female breast cancer in Denmark registered during the same 30-year period and similarly composed as to places and methods of treatment.

Most authors maintain that males with breast cancer have a much less favourable prognosis than women. However, many authors applied the observed survival rate in this comparison. When comparing the prognosis in male and female breast cancer, it is essential to consider two factors: (1) the mortality from other causes is higher in males than in females of the same age; (ii) the average age in the male patients is higher than that seen in the females. Therefore, only the relative, or corrected, survival rates can be compared. Table I shows that the 5-year corrected survival rate is as high as 60% in unselected series of male breast cancer. Moss (1964), MacKay and Sellers (1965), and Mausner *et al.* (1969) compared corrected survival rates for unselected series of female breast cancer registered during the same period and from the same treatment centres as the patients with male breast cancer. The 5-year corrected survival rates in the female patients were 56%, 50% and 63% respectively. Because of the rather limited numbers of cases in the series of male breast cancer, the range of sampling variability is greater than in the series of female breast cancer. Hence, Moss (1964) considered the 5-year corrected survival rates in female patients to be only slightly better than in male patients, and Mausner *et al.* (1969) considered the corrected 5-year survival rates to be similar in female and male patients. In the series of MacKay and Sellers (1965), the survival rates by extent of disease in males did not differ significantly from those in women.

Hence, it must be concluded that, on the basis of the most recent comparisons of corrected 5-year survival rates in females and males with this disease, the prognosis in males is not much worse than the prognosis in females.

(e) By itself, the duration of symptoms is an inaccurate parameter for prognosis (Table III). Prognosis can be poor in a patient with a rapidly growing tumour and symptoms of short duration, and good in a patient with a slowly growing tumour and symptoms of long duration. However, this does not indicate that the duration of symptoms is insignificant in the prognosis in male breast cancer. It has previously been shown in this series that the longer the patients have had a tumour, the more advanced is the clinical stage (Scheike, 1973), and the influence of the clinical stage on the prognosis is shown in Table IV. One of the reasons for the possibly poorer prognosis in males with breast cancer might be that the duration of symptoms is considerably longer than in women.

(f) The clinical TNM classification is significantly related to the prognosis in all four stages (Table IV).

(g) There is a significant correlation between the histological grade of malignancy and prognosis (Fig. 4). A comparison between the present series and series of female breast cancer showed that there were relatively fewer cases in the poorly differentiated Group III in the present series (Visfeldt and Scheike, 1973). A possibly poorer prognosis in males with breast cancer cannot, on the basis of the present series, be ascribed to a higher histological grade of malignancy, but must rather be caused by a longer duration of disease, and the anatomy of the male breast with the sparse mammary tissue.

(h) Because of the selective processes which in some cases determine how the patients will be managed and because the number of patients within the groups of treatment compared is not very great, some of the comments on the influence of treatment must be taken with certain reservations. Radical mastectomy does not give consistently better results than simple mastectomy (Table VIII). Hence, simple mastectomy is perhaps to be preferred, since it has fewer post-operative complications and is less strenuous, which is an important factor considering that the patients are on an average 65 years old, 40% being 70 years or older. Most authors recommend radical mastectomy as the optimum treatment in male breast cancer (Somerville, 1952; Holleb, Freeman and Farrow, 1968). However, some authors maintain that simple mastectomy is the treatment of choice (Greening and Aichroth, 1965; Liechty *et al.*, 1967). In the series presented by these two groups of authors, simple mastectomy with or without radiotherapy gave just as good results as radical mastectomy with or without radiotherapy.

(i) Supplementary radiotherapy did not improve the results of treatment (Table VIII).

(j) The treatment of male breast cancer should be centralized as much as possible, since the disease is so uncommon (about 10–15 new cases in Denmark annually), and since treatment often has to be decided individually.

My thanks are due to Mr J. Nyboe, Cand.act., statistician at Rigshospitalet, Copenhagen, for his critical review of the statistical calculations.

### REFERENCES

Bloom, H. J. G. (1950) Further Studies on Prognosis of Breast Carcinoma. *Br. J. Cancer*, **4**, 347.

Bloom, H. J. G. & Richardson, W. W. (1957) Histological Grading and Prognosis in Breast Cancer. *Br. J. Cancer*, **11**, 359.

Chrichlow, R. W. (1972) Carcinoma of the Male Breast. *Surgery Gynec. Obstet.*, **134**, 1011.

Edwards, J. H. (1958) A Note on the Interpretation of $n \times 2$ Tables. *Br. J. prev. soc. Med.*, **12**, 141.

Greening, W. P. & Aichroth, P. M. (1965) Cancer of the Male Breast. *Br. J. Cancer*, **19**, 92.

Haagensen, C. D. (1972) *Diseases of the Breast.* 2nd Edn. Philadelphia: Saunders.

Holleb, A. J., Freeman, H. P. & Farrow, J. H. (1968) Cancer of the Male Breast—II. *N.Y. St. J. Med.*, **68**, 656.

International Union Against Cancer (1968) *TNM Classification of Malignant Tumours.* Geneva.

Liechty, R. D., Davis, J. & Gleysteen, J. (1967) Cancer of the Male Breast. *Cancer, N.Y.*, **20**, 1617.

MacKay, E. N. & Sellers, A. H. (1965) Breast Cancer at the Ontario Cancer Clinics, 1938–56: A Statistical Review. *Can. med. Ass. J.*, **92**, 647.

Mausner, J. S., Shimkin, M. B., Moss, N. H. & Rosemund, G. P. (1969) Cancer of the Breast in Philadelphia Hospitals 1951–64. *Cancer, N.Y.*, **23**, 260.

Moss, N. H. (1964) Cancer of the Male Breast. *Ann. N.Y. Acad. Sci.*, **114**, 937.

Norris, H. J. & Taylor, H. B. (1969) Carcinoma of the Male Breast. *Cancer, N.Y.*, **23**, 1428.

Peltokallio, P. & Kalima, T. V. (1969) Malignant Tumours of the Male Breast in Finland. A Report of 51 Cases. *Br. J. Cancer*, **23**, 480.

Scarff, R. W. & Torloni, H. (1968) *Histological Typing of Breast Tumours.* (International Histological Classification of Tumours, No. 2). Geneva: WHO.

SCHEIKE, O. (1973) Male Breast Cancer. 5. Clinical Manifestations in 257 Cases in Denmark. *Br. J. Cancer*, **28**, 552.

SCHEIKE, O., SVENSTRUP, B. & FRANDSEN, V. A. (1973*a*) Male Breast Cancer. 2. Metabolism of Oestradiol-17$\beta$ in Men with Breast Cancer. *J. steroid. Biochem.*, **4**, 489.

SCHEIKE, O., VISFELDT, J. & PETERSEN, B. (1973*b*) Male Breast Cancer. 3. Breast Carcinoma in Association with the Klinefelter Syndrome. *Acta path. microbiol. scand.*, **81A**, 352.

SCHEIKE, O. & VISFELDT, J. (1973) Male Breast Cancer. 4. Gynecomastia in Patients with Breast Cancer. *Acta path. microbiol. scand.*, **81A**, 359.

SMITHERS, D. W., RIGBY-JONES, P., GALTON, D. A. G. & PAYNE, P. M. (1952) Cancer of the Breast. *Br. J. Radiol.*, Suppl. 4.

SOMERVILLE, P. (1952) Carcinoma of the Male Breast. A Report of 19 Cases and a Review of the Literature. *Br. J. Surg.*, **39**, 296.

TREVES, N. (1959) The Treatment of Cancer, Especially Inoperable Cases of the Male Breast by Ablative Surgery (Orchiectomy, Adrenalectomy and Hypophysectomy) and Hormone Therapy (Estrogens and Corticosteroids). An Analysis of 42 Patients. *Cancer, N.Y.*, **12**, 820.

TRISKA, H. (1967) Das Brustdrusenkarzinom beim Manne. Mitteilungsdienst G.B.K. NRWe. V., **4**, 535.

VISFELDT, J. & SCHEIKE, O. (1973) Male Breast Cancer. 1. Histologic Typing and Grading of 187 Danish Cases. *Cancer, N.Y.*, **32**, 985.

WILLIAMS, I. G. (1942) Carcinoma of the Male Breast. *Lancet*, ii, 701.